# The Relationship between Alexithymia, Dysmorphic Concern, and Exercise Addiction: The Moderating Effect of Self-Esteem

**DOI:** 10.3390/jpm11111111

**Published:** 2021-10-29

**Authors:** Alessio Gori, Eleonora Topino, Caterina Pucci, Mark D. Griffiths

**Affiliations:** 1 Department of Health Sciences, University of Florence, Via di San Salvi 12, Pad. 26, 50135 Florence, Italy; caterina.pucci@stud.unifi.it; 2 Integrated Psychodynamic Psychotherapy Institute (IPPI), via Ricasoli 32, 50122 Florence, Italy; 3 Department of Human Sciences, LUMSA University of Rome, Via della Traspontina 21, 00193 Rome, Italy; eleonora.topino@gmail.com; 4 Psychology Department, Nottingham Trent University, 50 Shakespeare Street, Nottingham NG1 4FQ, UK; mark.griffiths@ntu.ac.uk

**Keywords:** exercise addiction, alexithymia, emotional dysregulation, body image, self-esteem

## Abstract

As with other addictions, exercise addiction can severely impact individuals’ lives and have significant psychophysical consequences. Consequently, the study of the mechanisms involved in this psychopathological condition has great clinical and practical relevance. Therefore, the goal of the present study was to explore the risk factors and protective factors for exercise addiction, with a particular focus on the roles of alexithymia, body image concerns, and self-esteem. A sample of 288 regular exercisers (mean age = 28.35 years, SD = 8.26; 72% females, 18% males) completed the Exercise Addiction Inventory, 20-Item Toronto Alexithymia Scale, Body Image Concern Inventory, and Rosenberg Self-Esteem Scale. Data were analyzed by implementing a moderated mediation model. Results showed a significant and positive association between alexithymia and exercise addiction, totally mediated by body image concerns. Furthermore, self-esteem showed a relevant moderating effect, such that at high levels of self-esteem the effect of alexithymia on body image concerns became insignificant. Such data have important implications, highlighting some core variables on which it might be useful to keep a focus in order to elaborate tailored interventions, from both preventive and treatment perspectives.

## 1. Introduction

A large body of research has shown that physical activity—defined as any body movement that requires energy expenditure [1]—has positive effects on physical and mental health [2]. Recommendations for the minimum level of physical activity that is needed for beneficial health effects have been provided by the World Health Organization [3], with parameters based on age and physical condition. Specifically, the recommendation for people aged 18–64 years without chronic conditions or disabilities, and not in a pre/postpartum state, is at least 150–200 min of moderate-intensity aerobic physical activity, at least 75–150 min of vigorous-intensity aerobic physical activity, or an equivalent combination of these activities, per week [3]. However, when exercise becomes excessive, it can lead to the implementation of compulsive training patterns that can evolve into a pathology—so-called “exercise addiction” [4].

Some authors define exercise addiction as a morbid behavior in which individuals gradually lose control over their exercise habits, act compulsively, and experience negative consequences—both physically, and in their social and/or professional lives [5,6]. Physical damage is manifested predominantly through long-term risks such as musculoskeletal injuries and psychological damage (typically expressed through immediate changes in mood, such as the feeling of depression when the individual cannot exercise) [6,7]. More specifically, the symptoms and consequences of exercise addiction have been characterized by six common components of addiction: salience, mood modification, tolerance, withdrawal symptoms, personal conflict, and relapse [8,9]. In the event of addiction, the negative consequences are ignored, and do not prevent individuals from continuing to exercise [9,10]. Investigations of these factors have been helpful to the field from both preventive and treatment perspectives [11,12]. Therefore, the present study explores the interaction of risk factors and protective factors in pathways towards exercise addiction among regular exercisers, with a particular focus on the roles of alexithymia, body image concerns, and self-esteem.

Alexithymia, a construct originally introduced by Sifneos [13], is a specific form of emotional dysregulation that is defined by a difficulty in identifying, describing, and verbalizing emotions, as well as difficulty by individuals in discriminating their own emotional experiences from underlying physiological activation, which is also characterized by constricted imaginary processes [14,15,16]. It appears that psychic suffering resulting from affective dysregulation can lead those with alexithymia to regulate their negative emotions through behaviors that may be a risk to their health (e.g., drug use, engaging in risky sports, eating disorders) [17,18,19]. Therefore, it is not surprising that many scientific studies have shown a relationship between alexithymia and substance dependence [17,20] and behavioral addictions [21,22,23], including that related to exercise addiction.

The relationship between alexithymia and exercise addiction has been examined in different populations of exercisers, such as those attending fitness centers [24], swimmers [25], and sports university students [26]. Some research has suggested that the use of physical activity could be a means by which these individuals try to suppress their unmentalized emotional states, as a form of dissociation from painful experiences [17,24]. An interesting point of view is provided by some research arguing that the inability to symbolize emotional experiences—as well as the resulting undifferentiated and dysregulated affect—may also lead to body image distortion [27]. In other words, the inability of an individual to discriminate between emotional states and bodily sensations can increase dissatisfaction with their body, and could lead to a wrong interpretation of the perceptual and behavioral aspects of their body image [28]. Such inability could indeed arouse an emotional void that leads individuals to focus excessively on the details of their own body. This, in turn, can result in the use of maladaptive strategies (including excessive exercise) in order to control the body and physical appearance [26], as found in some cases of body dysmorphic disorder (BDD) [27].

On this basis, it is important to emphasize the associations between body concerns and exercise addiction. Several studies (e.g., [29,30]) have found different psychopathological conditions that co-occur with exercise addiction; among these, BDD is a severe psychiatric condition characterized by a recurring and persistent concern with an imagined or minor defect in physical appearance, with a focus on a specific body part [29]. In fact, individuals showing excessive concern over body image and weight are among those most likely to experience exercise addiction [31]. Some other studies have suggested that preoccupation with body image may be a driving force underlying exercise addiction [32]. Indeed, this dissatisfaction may lead to the search for a transformation of the perceived body image into an “ideal body image”, through inadequate nutritional planning and excessive physical exercise. Consequently, physical exercise could become a vehicle to improve body image, as opposed to being motivated by the desire for increased health and wellbeing [29].

Within this framework, self-esteem could also be a relevant factor, since lower levels of self-esteem can influence the ways individuals perceive their own bodies [33,34]. Scientific literature agrees that self-esteem plays a central role in individuals’ mental health, and it is more likely that a positive self-image and a strong sense of self-esteem help individuals to become more satisfied with their bodies [35]. In fact, higher self-esteem may protect the individual from the negative feelings related to their body weight, and from anxiety arising from the negative judgments of others [36,37,38], showing a beneficial influence on body-image-related preoccupation [35,39]. On the other hand, individuals with low self-esteem appear to be more vulnerable to comments concerning their bodies, and more dissatisfied with physical aspects of their bodies over time [36,40]. Previous evidence suggests that increased self-esteem could play a protective role against body image concerns [41].

**Figure 1:**
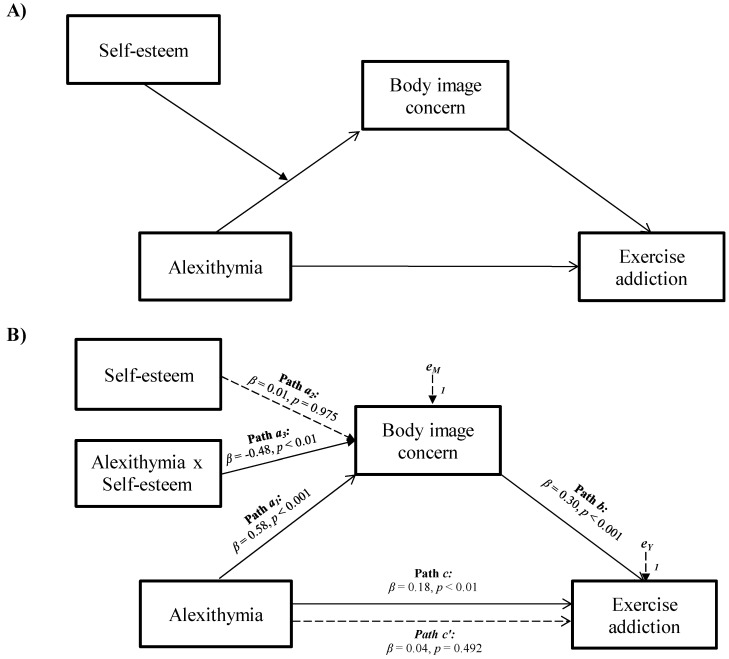
Statistical (**A**) and conceptual (**B**) forms of the moderated mediation model involving alexithymia, body image concerns, self-esteem, and exercise addiction.

Given this evidence, the present study examined the role of alexithymia, body image concerns, and self-esteem in exercise addiction, by testing a moderated mediation model among a sample of regular exercisers. More specifically, it was hypothesized that body image concerns would mediate the relationship between alexithymia and exercise addiction, with self-esteem moderating the relationship between alexithymia and body image concerns (see Figure 1A).

## 2. Materials and Methods

### 2.1. Participants, Procedure, and Ethics

The sample comprised 288 Italian participants who declared that they regularly engaged in exercise (i.e., at least three times per week for a minimum of 30 min each session). Their age ranged from 19 to 53 years (*M_age_* = 28.35 years, *SD* = 8.26), and they were predominantly females (72%). As shown in Table 1, most of the participants declared that they were single (75%), had a high school diploma (46%), and were students (35%).

All of the participants were recruited online. The study was advertised on the authors’ various social networks, with a recruitment message that included an anonymous link to the survey. Therefore, the survey was further distributed utilizing a snowball sampling method. The participants voluntarily took part in the study by completing a self-report survey hosted on the *Google Forms* platform, after they had been informed about the general aims of the research and provided informed consent electronically. The protocol of the present study was approved by the Ethical Committee of the Integrated Psychodynamic Psychotherapy Institute (IPPI) (ethical approval number 004/2021).

### 2.2. Measures

*Exercise Addiction Inventory* (EAI): The EAI [9,42] is a self-report measure that assesses the risk of exercise addiction. The six items (e.g., “*If I have to miss an exercise session, I feel moody and irritable*”) were developed using the components model of behavioral addiction (Griffiths, 1996), and comprise the dimensions of salience, mood modification, tolerance, withdrawal symptoms, conflict, and relapse. Items are scored on a five-point Likert scale from 1 (*strongly disagree*) to 5 (*strongly agree*). The total scores range from 6 to 30, with higher scores indicating more problematic exercise for the individual. A cutoff score for individuals considered at risk of exercise addiction is 24, while a score of 13–23 indicates a symptomatic individual, and a score of 0–12 suggests an asymptomatic individual [9]. Cronbach’s alpha value for the Italian version [43] used in the present study was *α* = 0.71.

*Twenty-Item Toronto Alexithymia Scale(TAS-20): The* TAS-20 [14,15] is a self-report measure that assesses alexithymia. The 20 items of the TAS-20 are scored on a five-point Likert scale from 1 (“*strongly disagree*) to 5 (*strongly agree*) and comprising three subscales: difficulty identifying feelings (e.g., “*I am often confused about what emotion I am feeling*”), difficulty describing feelings (e.g., “*It is difficult for me to find the right words for my feelings*”), and externally oriented thinking (e.g., “*I prefer to analyze problems rather than just describe them*”). Cronbach’s alpha value for the Italian version [44] used in the present study was *α* = 0.75 for the total scale.

*Body Image Concern Inventory* (BICI): The BICI [45] is a self-report measure that assesses dysmorphic body image concerns. The 19 items of the BICI are scored on a five-point Likert scale from 1 (*never*) to 5 (*always*) and comprising two subscales: dysmorphic symptoms (e.g., “*I am dissatisfied with some aspect of my appearance*”), and symptom interference (e.g., “*I have missed social activities because of my appearance*”). Cronbach’s alpha value for the Italian version [46] used in the present study was *α* = 0.91 for the total scale.

*Rosenberg Self-Esteem Scale* (RSES): The RSES [47] is a self-report measure that assesses self-esteem. The 10 items of the RSES are scored on a four-point Likert scale from 0 (*strongly disagree*) to 3 (*strongly agree*). Cronbach’s alpha value for the Italian version [48] used in the present study was *α* = 0.84.

### 2.3. Data Analysis

Data were analyzed using SPSS for Windows (v. 21). A two-sided value of *p* < 0.01 was the level of statistical significance in the present study. There were no missing values in the dataset because the online platform used did not allow the submission of surveys unless all items were answered. Descriptive statistics for the sample and the study measures were carried out. A Pearson’s *r* correlation analysis was performed to investigate the associations between the variables, together with the coefficient of determination (R^2^). According to Cohen [49], values of 0.25, 0.09, and 0.01 correspond to large, moderate, or small relationships, respectively. The hypothesized moderated mediation model was tested through the macro-program PROCESS 3.4 [50], by performing Model 7. For completeness, the potential confounding role of age was also explored in the model. The 95% confidence interval (CI) was calculated for each regression coefficient, such that when the 95% CI (from LLCI to ULCI) does not contain the zero, the effect should be considered significant. The conditional indirect effect was evaluated following Wayne et al.’s [51] procedure, by analyzing the index of the moderated relationship at three different levels of the moderator (−1DS, Mean, +1DS). Furthermore, a bootstrapping procedure with 95% CI at 5000 samples was used to confirm the statistical significance of the moderation effect. When the bootstrapped confidence interval (from boot LLCI to boot ULCI) does not contain the zero, the effect should be considered significant.

## 3. Results

Descriptive statistics are reported in Table 1 and Table 2. Pearson’s *r* analysis (see Table 2) showed that the highest correlation was between exercise addiction and body image concern (*r* = 0.317, *p < 0*.01), explaining 30% of the variance. Furthermore, there were significant positive correlations between exercise addiction and age (*r =* 0.153, *p* < 0.01) and exercise addiction and alexithymia (*r* = 0.178, *p <* 0.01). There was a significant negative correlation between exercise addiction and self-esteem (*r* = −0.152, *p <* 0.01). In turn, self-esteem was significantly negatively correlated with body image concerns (*r* = −0.608, *p <* 0.01) and alexithymia (*r* = −0.512, *p <* 0.01) scores. Body image concerns and alexithymia were significantly positively correlated (*r* = 0.454, *p <* 0.01).

The moderated mediation analysis showed that body image concerns totally mediated the relationship between alexithymia and exercise addiction, and the association between alexithymia and body image concerns was moderated by self-esteem (see Figure 1).

More specifically, the total effect of alexithymia on exercise addiction was significant and positive (Path *c* in Figure 1B; *β* = 0.18, *p <* 0.01, LLCI = 0.0217 − ULCI = 0.0997). Alexithymia was also significantly and positively associated with body image concerns, the mediator variable (Path *a*_1_ in Figure 1B; *β* = 0.58, *p <* 0.001, LLCI = 0.3846 − ULCI = 1.1129). Body image concerns showed a significant and positive relationship with exercise addiction (Path *b* in Figure 1B; *β* = 0.30, *p <* 0.001, LLCI = 0.0458–ULCI = 0.1115) and, when included in the model, totally mediated the association between alexithymia and exercise addiction (see Model 1a in Table 3), which became insignificant (Path *c’* in Figure 1B; *β* = 0.04, *p* = 0.492, LLCI = −0.0275 − ULCI = 0.0570). Furthermore, self-esteem was found to be a significant moderator in the relationship between alexithymia and body image concerns (Path *a*_3_ in Figure 1B; *β* = −0.48, *p <* 0.01, LLCI = −0.0413 − ULCI = −0.0079): Δ*R*^2^ = 0.017, *F*(1, 285) = 6.810, *p <* 0.01 (index of moderated mediation = −0.0019, Boot LLCI = −0.0037 − Boot ULCI = −0.0007). 

**Figure 2:**
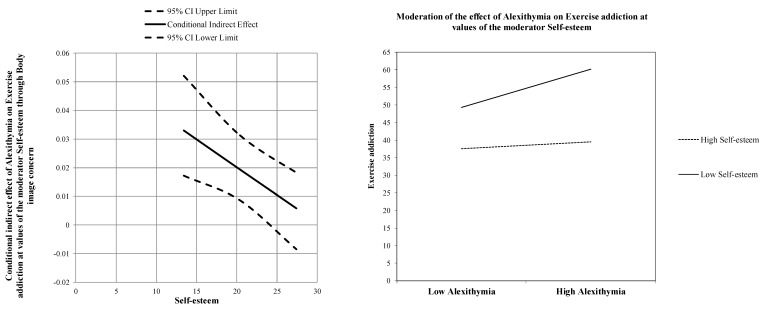
Graphical representation of the moderation effect.

The conditional indirect effect was evaluated by analyzing the index of the moderated relationship at three different levels of the moderator (−1DS, Mean, +1DS). The association between alexithymia and body image concerns was slightly stronger at low levels of self-esteem (estimate = 0.419[0.10], *p <* 0.001, LLCI = 0.2425 − ULCI = 0.5958) than at average levels (estimate = 0.246[0.07], *p <* 0.001, LLCI = 0.1131 − ULCI = 0.3806), and became insignificant at high levels (estimate = 0.075[0.09], *p =* 0.822, LLCI = −0.1039 − ULCI = 0.2529). Therefore, when participants reported higher levels of self-esteem, the positive indirect effect of alexithymia on exercise addiction via body image concerns weakened to become insignificant: effect = 0.0059(0.0068), BootLLCI = −0.0085- BootULCI = 0.0183 (see Figure 2).

Finally, the statistical significance of the moderation effect was confirmed via the bootstrapping procedure, since the bootstrapped confidence interval did not contain the zero: Boot LLCI = −0.0421 − Boot ULCI = −0.0098.

The potential confounding of age was also examined. Age showed a significant covariant effect on exercise addiction for both the indirect effect of alexithymia on exercise addiction via the mediation of body image concern, and the moderation of body image concern (*β* = 0.20, *p* < 0.001, Boot LLCI = 0.0547 − Boot ULCI = 0.1594), as well as for the total effect (*β* = 0.19, *p* < 0.01, Boot LLCI =0.0444–Boot ULCI = 0.1621). Moreover, controlling for age, both the total effect of alexithymia on exercise addiction (*β* = 0.21, *p* < 0.001, LLCI = 0.0331 − ULCI = 0. 1110) and the moderated mediation model (see Model 1b in Table 3) remained statistically significant.

## 4. Discussion

The physical, psychological, aesthetic, and social benefits of regular exercise activity are well documented [52,53,54], both for the adult population [54] and in the pre-adult developmental phase [55]. However, evidence is emerging in the literature that for a small minority of individuals, excessive physical exercise can acquire the features of an addiction [12,56], characterized by feelings of loss of control, overtraining problems such as fatigue and sleep disturbances, and withdrawal symptoms such as restlessness, sadness, and irritability [57]. In light of these considerations, and the psychophysical damage associated with this condition, the study of the mechanisms involved in the development and maintenance of this unhealthy form of exercise acquires great clinical and practical relevance. Given this framework, the present research analyzed the interaction between alexithymia, body image concerns, and self-esteem in contributing to exercise addiction among regular exercisers.

First, our results showed a significant and positive influence of alexithymia on exercise addiction, concurring with previous research [58,59]. This is consistent with other evidence highlighting the role of alexithymia and, more generally, emotional dysregulation in facilitating addictive behaviors, which may become dysfunctional strategies to cope with painful emotions (see Morie et al. [18] for a review). Indeed, given their lack of emotional awareness, individuals with high levels of alexithymia tend to have difficulty in managing their affect [14,60,61], and engaging in addictive behavior may become a dysfunctional strategy to cope with painful emotion [62,63]. Consistently, the theory of affect regulation [64] suggests that physical activity may lead to improvements in positive moods and decreases in negative ones (anxiety, irritability, and guilt). Therefore, some individuals may consider exercise as a means of coping with stress, to the point of becoming addicted to it [56,64,65,66]. Furthermore, the findings of the present study showed a significant positive association between alexithymia and body image concerns.

Consistently, previous evidence has found that poor emotional expression is related to higher levels of body dissatisfaction, among both clinical [27] and nonclinical samples [28]. One of the characteristics of alexithymia is that individuals have difficulty in understanding their own affective experiences and/or the association between emotional states and somatic manifestations. This may lead to an excessive focus on physical components and body image distortions so as to avoid contact with the emotional experiences [28,67]. Our results also highlighted the significant and positive influence of body image concerns on exercise addiction, determining a total mediation in the relationship between alexithymia and exercise addiction. Consistent with these data, significant and positive associations between negative body image and pathological exercise behaviors have previously been found [42,68].

This can be understood in light of the great potential that exercise has to modify the characteristics of the body (see Marques et al. [69] for a review). Therefore, body dissatisfaction may lead to morbid exercise, both through negative reinforcement (e.g., guilt by individuals for wasting opportunities to improve their appearance when skipping workouts) and positive reinforcement (e.g., a more toned body) [70]. Therefore, by integrating and enriching the existing evidence, data from the present research suggested that the total effect of alexithymia on exercise addiction did not occur directly, but manifested itself indirectly through the increase in dissatisfaction related to an individual’s own body image, which resulted in morbid exercise, plausibly interpretable as a dysfunctional coping strategy [65]. However, the results also showed the relevant influence of self-esteem in this indirect path, so that as the score of self-esteem increased, the effect of alexithymia on body image concerns diminished to become insignificant. Such data are consistent with a previous study that highlighted an inverse relationship between self-esteem and negative perceptions of body image [28], and further corroborate the existing evidence relating to the core role of self-esteem in psychological wellbeing (e.g., [71]) and as a protective factor for mental health (e.g., [72]).

Additionally, age was found to be a significant confounding variable in the model, given its effect on exercise addiction. More specifically, older participants reported higher levels of morbid physical activity. These data add to the extant literature that currently reports conflicting results, sometimes identifying higher levels of exercise addiction among young people, while other studies report no difference based on age (e.g., [73,74]). Further studies are needed in order to investigate this aspect.

The present study has some limitations, which should be noted when interpreting its findings. First, the cross-sectional design of this research hinders the inference of causal links between the variables under examination. Furthermore, the implementation of the moderated mediation model did not consider the bidirectionality of the associations between the variables. Although the hypothesized links were based on a solid body of pre-existing literature, the present data only provide preliminary support for the observed relationships. Future longitudinal research is needed in order to test the relationships empirically, as well as considering the possibility of bidirectionality in the association between the variables, and further enriching the model by exploring the roles of other promising factors in the field of addiction and mental health, such as attachment (e.g., [75]), family functioning (e.g., [76]), and dissociation (e.g., [61]), to name but a few. Furthermore, data were collected online, and this could limit the generalizability of the study (for example, with respect to exercisers who did not have internet access). The relatively small sample size should also be noted when interpreting the study’s outcomes. Moreover, the imbalance concerning some demographic variables (e.g., gender, relationship status, occupation) did not allow reliable evaluation of their relationship with the risk/protective factors included in the model, or of the relationships between them. Therefore, a more in-depth study utilizing a larger and more balanced sample, recruited with a more extensive and representative sampling method, is needed for future research, in order to provide a more complete picture of these results. In addition, no information was collected on the level of physical activity (e.g., recreational or competitive). Future research could explore the influence of this variable on the hypothesized moderated mediation model. Finally, self-report measures were used, and this exposed the possibility of some bias (e.g., social desirability). Integration of other kinds of measure (e.g., structural interviews) following a multimethod approach could be important in future research to overcome this limitation. Therefore, in light of these limitations, the results must be generalized with caution, and studies with more representative samples of the national population, with a better distribution for demographic variables (e.g., gender, occupational status, relational status, etc.), are necessary. Further research is also needed examining the level/mode of sport (competitive/non-competitive; individual/team sport), with a longitudinal design and an integrated multimethod collection of further variables of interest, in order to obtain a clearer picture. On the other hand, the present research offers preliminary data that provide useful indications concerning the protective value of high levels of self-esteem against exercise addiction, as well as the importance of considering the potential risks that could be associated with alexithymia and body image concern.

## 5. Conclusions

As with other addictions, exercise addiction influences individuals in their daily lives, resulting in a loss of control and psychophysical damage [70]. Given the significant impairment in the lives of affected individuals, increasing research has focused on risk factors and antecedents for exercise addiction (e.g., [77]). Within this framework, the present study explored the positive relationships between alexithymia, body image concerns, and exercise addiction, but also confirmed the important protective role of self-esteem. These findings provide wider knowledge and insight regarding the variables associated with exercise addiction, and may have important clinical implications—for example, by orienting preventive activities among regular exercisers, as well as addressing tailored treatments for addicted individuals.

## Figures and Tables

**Table 1:** Demographic characteristics of the sample (*N =* 288).

Characteristics		*M* ± *SD*	*n*	%
*Age* (years)		28.4 ± 8.26		
*Sex*				
	Females		206	71.5
	Males		82	28.5
*Marital Status*				
	Single		215	74.7
	Married		25	8.7
	Cohabiting		39	13.5
	Separated		4	1.4
	Divorced		4	1.4
	Widowed		1	0.3
*Education*				
	Middle school diploma		8	2.8
	High school diploma		132	45.8
	University degree		86	29.9
	Master’s degree		43	14.9
	Post-lauream specialization		19	6.6
*Occupation*				
	Student		98	34.0
	Working student		58	20.1
	Employee		78	27.1
	Freelance		18	6.3
	Entrepreneur		12	4.2
	Trader		8	2.8
	Artisan		3	1.0
	Armed forces		1	0.3
	Unemployed		11	3.8
	Retired		1	0.3

**Table 2:** Pearson’s correlation, means, and standard deviations of the study variables.

	1	2	3	4	5	6	7	8	9
1. EAI	1								
2. TAS-20	0.178 **	1							
(R^2^)	(0.032)								
3. TAS-20 (F1)	0.163 **	0.803 **	1						
(R^2^)	(0.027)	(0.645)							
4. TAS-20 (F2)	0.214 **	0.844 **	0.556 **	1					
(R^2^)	(0.046)	(0.712)	(0.309)						
5. TAS-20 (F3)	0.006	0.585 **	0.279 **	0.211 **	1				
(R^2^)	(0.000)	(0.342)	(0.078)	(0.045)					
6. RSES	−0.152 **	−0.512 **	−0.397 **	−0.559 **	−0.137 *	1			
(R^2^)	(0.023)	(0.262)	(0.158)	(0.312)	(0.019)				
7. BICI	0.317 **	0.454 **	0.331 **	0.538 **	0.091	−0.608 **	1		
(R^2^)	(0.100)	(0.206)	(0.110)	(0.289)	(0.008)	(0.370)			
8. BICI (F1)	0.305 **	0.449 **	0.343 **	0.531 **	0.078	−0.598 **	0.990 **	1	
(R^2^)	(0.093)	(0.202)	(0.118)	(0.282)	(0.006)	(0.358)	(0.980)		
9. BICI (F2)	0.298 **	0.382 **	0.218 **	0.457 **	0.126 *	−0.525 **	0.837 **	0.751 **	1
(R^2^)	(0.089)	(0.146)	(0.048)	(0.209)	(0.016)	(0.276)	(0.701)	(0.564)	
10. Age	0.153 **	−0.175 **	−0.151 *	−0.173 **	−0.073	0.191 ^**^	−0.104	−0.128 *	0.012
(R^2^)	(0.023)	(0.031)	(0.023)	(0.030)	(0.005)	(0.036)	(0.011)	(0.016)	(0.000)
*M*	17.510	47.656	13.736	16.618	35.014	20.406	47.781	40.903	6.878
*SD*	4.446	13.061	5.091	6.924	4.401	7.006	16.795	13.927	3.595

***Note:*** Bold values indicate significant *p*-values. **: Correlation is significant at the *p* *<* 0.01 level (2-tailed); *: correlation is significant at the *p* < 0.05 level (2-tailed). EAI: Exercise Addiction Inventory; TAS-20: 20-Item Toronto Alexithymia Scale; TAS-20 (F1): difficulty describing feelings (20-Item Toronto Alexithymia Scale); TAS-20 (F2): difficulty identifying feelings (20-Item Toronto Alexithymia Scale); TAS-20 (F3): externally oriented thinking (20-Item Toronto Alexithymia Scale); RSES: Rosenberg Self-Esteem Scale; BICI: Body Image Concern Inventory; BICI (F1): dysmorphic symptoms (Body Image Concern Inventory); BICI (F2): symptom interference (Body Image Concern Inventory).

**Table 3:** Coefficients of the models.

Model 1a										
		Consequent								
		M (Body image concern)					Y (Exercise addiction)			
Antecedent		Coeff.	SE	*p*	95% CI		Coeff.	SE	*p*	95% CI
X (Alexithymia)	*a*_1_	0.749	0.185	0.001	[0.385, 1.113]	*c’*	0.015	0.022	0.492	[−0.028, 0.057]
M (Body image concern)		-	-	-	-	*b*	0.079	0.017	< 0.001	[0.046, 0.112]
W (Self-esteem)	*a*_2_	0.014	0.443	0.975	[−0.859, 0.886]		-	-	-	-
X * W	*a*_3_	−0.025	0.009	0.004	[−0.041, −0.008]		-	-	-	-
Constant	*i_M_*	34.590	19.389	0.004	[14.141, 55.040]	*i_Y_*	13.051	1.001	< 0.001	[11.081, 15.021]
		*R*^2^ = 0.415 *F*(3, 284) = 67.190, *p <* 0.001					*R*^2^ = 0.102 *F*(2, 285) = 16.154, *p <* 0.001			
**Model 1b**										
		Consequent								
		M (Body image concern)					Y (Exercise addiction)			
Antecedent		Coeff.	SE	*p*	95% CI		Coeff.	SE	*p*	95% CI
X (Alexithymia)	*a*_1_	0.749	0.185	0.001	[0.385, 1.113]	*c’*	0.015	0.022	0.492	[−0.016, 0.068]
M (Body image concern)		-	-	-	-	*b*_1_	0.080	0.016	< 0.001	[0.048, 0.112]
W (Self-esteem)	*a*_2_	−0.002	0.445	0.975	[−0.878, 0.874]		-	-	-	-
X * W	*a*_3_	−0.024	0.009	0.004	[−0.041, −0.008]		-	-	-	-
C (Age)	*a*_4_	0.050	0.095	0.601	[−0.137; 0.236]	*b*_2_	0.106	0.030	< 0.001	[0.048; 0.112]
Constant	*i_M_*	33.363	10.663	0.020	[12.373, 54.353]	*i_Y_*	9.440	1.416	< 0.001	[6.653, 12.227]
		*R*^2^ = 0.416 *F*(4, 283) = 50.332, *p <* 0.001					*R*^2^ = 0.140 *F*(3, 284) = 15.375, *p <* 0.001			

***Note:*** Model 1a: the relationship between alexithymia and exercise addiction, with body image concern as mediator and self-esteem as moderator; Model 1b: the relationship between alexithymia and exercise addiction, with body imageconcern as mediator, self-esteem as moderator, and age as covariate.

## Data Availability

The data presented in this study are available on request from the corresponding author. The data are not publicly available, for reasons of privacy.
